# A smartphone-based consultation system for acute burns – methodological challenges related to follow-up of the system

**DOI:** 10.1080/16549716.2017.1328168

**Published:** 2017-08-25

**Authors:** Marie Hasselberg, Lee Wallis, Paul Blessing, Lucie Laflamme

**Affiliations:** ^a^ Department of Public Health Sciences, Karolinska Institutet, Stockholm, Sweden; ^b^ Stellenbosch Institute for Advanced Study (STIAS), Wallenberg Research Centre at Stellenbosch University, Stellenbosch, South Africa; ^c^ Division of Emergency Medicine, Faculty of Medicine and Health Sciences, Stellenbosch University, Bellville, South Africa; ^d^ Institute of Social and Health Sciences, University of South Africa, Pretoria, South Africa

**Keywords:** mHealth for Improved Access and Equity in Health Care, Acute burns, mHealth, burn outcome measures, image-based diagnostic

## Abstract

**Background**: A smartphone-based consultation system for acute burns is currently being implemented in the Western Cape, South Africa. Even though studies indicate that similar systems for burns tend to support valid diagnosis and influence patient management, the evidence is still sparse. There is a need for more in-depth evaluations, not least in resource-constrained settings where mHealth projects are increasing.

**Objective**: This article describes the consultation system and assessments in relation to its implementation with a special focus on methodological challenges.

**Methods**: A number of evaluations and assessments have been conducted, are ongoing or planned for in relation to the implementation of the teleconsultation system. Initial assessments showed that size and depth of burns could be assessed at least as well using photographs as at bedside and that the image quality of handheld devices can be used as well as computers. Studies on system usability are currently being done with a mixed-methods approach. A historical cohort design will be applied to assess the potential health impact of the system. Patients with burn injuries where the doctor at point of care has used the app to receive diagnostic support from a burns expert will be considered as exposed and patients with burn injuries where the app has not been used will be considered as non-exposed.

**Conclusions**: Smartphone-based consultation systems have the potential to strengthen the assessment of burn injury in many settings. However, ethically and methodologically sound evaluations are needed to find the best systems and solutions. This article identifies challenges and suggests potential assessments in relation to the implementation of such a system.

## Background

The use of telemedicine has relatively recently expanded in emergency medicine [1–4]. When a front-line clinician at a local emergency department requires specialist advice on the management of a patient, telemedicine is a very useful communication facilitator. In particular the use of mobile phone-based applications (mHealth) allows consultants to access pictures and real-time patient data to make more targeted and better-informed clinical decisions [1–4].

An area where mHealth has been introduced is burn injuries [4–8], which are estimated to account for at least 265,000 deaths globally each year and are largely attributable to poor living conditions [9]. The improvements seen in burn injury prevention and care over recent decades such as improved design of cook stoves, safety regulations covering housing design and materials, fire safety education and the use of smoke detectors have mainly benefitted those living in high-income countries, and burn mortality still remains unacceptably high in low- and middle-income countries [10]. Timely care is a prerequisite to reduce burn-related morbidity and mortality and mHealth has the potential to significantly support the provision of timely and appropriate care in resource-constrained settings [7]. Burn injuries are often difficult to assess by non-specialists and the relatively superficial and visual nature of burn injuries makes them a suitable target for mHealth applications [5]. A recent audit from South Africa showed that two thirds of the referrals were changed when photographs were added to the ordinary telephonic referral [7].

Even though studies indicate that image-based teleconsultation systems for burns tend to support valid diagnosis and can influence patient management, the evidence is still sparse and mainly derived from pictures taken with a digital camera and images assessed on a computer screen [4–8]. The technological quality of smartphone cameras has developed and improved quickly and smartphones are now used both for taking pictures and for expert assessments of images.

There are a number of aspects that need to be further evaluated in relation to the implementation of image-based mHealth systems and to assess the potential health impact, especially in resource-constrained settings. For that purpose, a randomized controlled trial is commonly seen as the gold standard. However, the design has a range of ethical and practical implications. First, the time lag between recruiting the patients and the outcome of the study in a randomized controlled trial may be critical since the technology might be out-of-date before the trial is finalized [11]. Secondly, it is debatable whether it is ethical to introduce randomization of a diagnostic support system in the acute phase of a burn. Thirdly, the cost of extensive data collection has to balance out the benefit of the system, especially in an already strained health system [12]. Alternative study designs that can be considered are interrupted time-series or historical cohort design [11].

A smartphone-based consultation system for acute burn injuries is currently being implemented in the Western Cape, South Africa. This article describes the system in question and assessments in relation to its implementation with a special focus on methodological challenges.

## A smartphone-based consultation system for acute burns

The study includes partners from South Africa, Sweden and Finland with a joint interest to increase health equity through improved access to care. It is taking place in Western Cape Province in South Africa – a diverse province consisting of greater than 5 million individuals, where more than 80% of the population is black or coloured and 15% is white [13]. The province contains both rural and urban areas with a wide range in socio-economic status. The study is taking place at all Western Cape emergency centres and burn referrals are sent to either Tygerberg Hospital (adults) or Red Cross Children’s Hospital (children), both tertiary hospitals in the Western Cape where the burn care specialists are on call.

The development of a smartphone-based consultation system for acute burns started in 2012 and from the beginning an app was installed on dedicated smartphones where structured patient data and photographs could be captured in a protocol-driven manner [14]. The project advanced in close collaboration with a panel of South African burns experts and emergency specialists and they soon requested that the app could be downloaded on their own smartphones. The original app has since the first version been changed and developed further by the Vula Mobile team and integrated into the Vula platform (www.vulamobile.com).

The Vula mobile app concerns the care of patients at the point of entry into the health care system: emergency care. An application for smartphones including clinical decision support is available at the health facilities to transmit visual and textual information between emergency doctors at point of care and a network of burns specialists. The application is already used by many front-line users in the Western Cape for referrals to ophthalmology, cardiology, orthopaedics and more, including a functionality for referring burn patients. Structured patient data and photographs can be captured in a protocol-driven manner in the client application.

Front-line users (clinicians, nurses) download the Vula mobile app onto their personal smartphones. The app is free of charge to download and is available on Android and iOS devices. Front-line users open the app, enter the patient data into text fields and upload de-identified images of the patient’s burn into the app. They can then send the referral along with a clinical question. The burn specialist on call is informed via instant messaging that a case has been uploaded and can then review the visually transmitted information and images and provide diagnostic and treatment advice. Referrals are transmitted to the burn care consultant or duty senior registrar at either Tygerberg Hospital or Red Cross Children’s Hospital in Cape Town. One of these individuals is on-site and able to receive referrals 24 hours a day. They are able to respond to referrals using a smartphone, PC or tablet. All of the information the front-line user entered is visible to the burns specialist, and they are able to interact with the front-line user via an SMS function within the app in real time. The app includes a server application that can store user information and de-identified patient data via the secure Vula cloud server.

Training programmes are offered to all facilities that use the app. Training sessions are 30 minutes long and focus on basic use of the application along with a test case. Staff training for using the system will be designed taking into consideration users’ different levels of experience with smartphones. Anxiety about using these kinds of devices is an issue that must be considered and the training should be designed to empower the staff and not intimidate. Continuous quality management is offered by the Vula Mobile team as well as by members of the research team. Technical problems are handled by the Vula support staff, while any issues with the study protocol or the referral process are handled by the research team. The app is routinely updated by the Vula Mobile team.

## Follow-up design and outcome measures

Formative evaluations in order to improve the app and its implementation process are ongoing but not described in detail here. The focus of this article is rather on previous and current evaluations related to image-based consultation and the health impact of the system.

**Figure 1.**
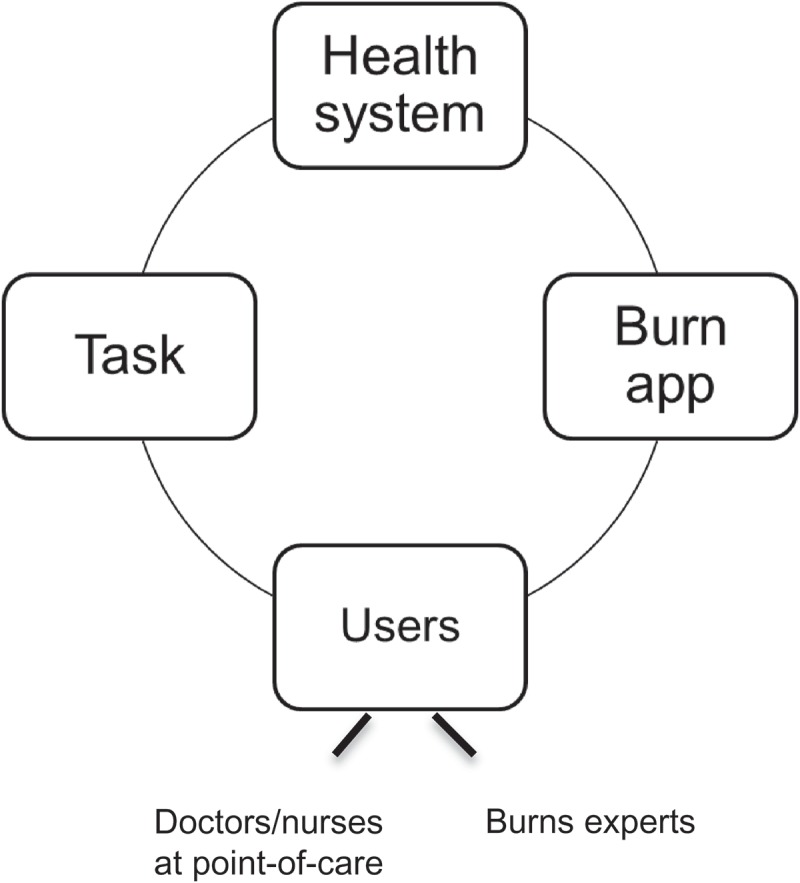
The organizational interaction diamond model, adapted from Leavitt’s Diamond model (1972) [15].

According to Leavitt’s Diamond model for organizational change, an organization has four components that are all interdependent and a change in one of them will also affect the other three (see Figure 1) [14]. For example, when introducing a new technology such as an app, people need to change their behaviour, they may need training to use the new technology and this may affect the health system.

### User perspective

One aspect of evaluation, often overlooked, is how the teleconsultation system is experienced by the users (front-line users and burns experts). Usability evaluation includes both perceived ease of use and perceived usefulness and can help with identifying whether the user encounters problems while using the system. Ongoing usability evaluations within the project have a mixed-methods approach. Semi-structured interviews have been conducted to capture the burns experts’ views on the consultation system. The experts were encouraging about the system and willing to adopt it. They agreed that the app could contribute to increased education opportunities and better ability to advise and diagnose [16].

Both front-line health care providers and burns experts has been invited to ‘think-aloud sessions’ where they express their thoughts, feelings and opinions while using the app. Furthermore, a structured questionnaire (Health-Information Technology Usability Scale [17]) will be disseminated when the app has been used more frequently.

### Image quality and diagnostic accuracy

The accuracy of image-based remote diagnostics by a medical expert is heavily dependent on the quality of the images at hand. At the start of this project, studies on image-based diagnostic accuracy had mainly been based on first-hand images taken with a camera (e.g. images of wounds) or images of clinical data like scans of radiologic results. There was therefore little knowledge concerning the expected quality of images taken at point of care with smartphone cameras, how experts would consider the quality of clinical images taken with smartphone cameras when viewing them either on a computer or on handled devices (like tablets or smartphones) and the expected diagnostic accuracy of images viewed on either computers or handled devices. A number of studies have therefore been undertaken to assess those questions with the following results.

#### Assessment of quality of images taken with smartphones when viewed by laypeople

This study considered how images (e.g. of objects, people or situations) are perceived by laypeople when taken by different smartphone cameras as opposed to being taken with a digital camera (all taken by a specialist photographer) [18]. The results showed wide variations among raters (60 people in total) for any given image and camera but a relatively good level of satisfaction. Incidentally one smartphone performed better than both the other smartphones and the digital camera.

#### Assessment of quality of images when viewed by experts on handheld devices

This study focused on whether images viewed on handheld devices were perceived by experts as comparable in quality to when viewed on a computer screen [19]. For that purpose, we used different types of images – of burns, skin lesions, radiology, scans, and a non-clinical one – and conducted a survey among clinicians with different backgrounds. We observed that images viewed on handheld devices, both smartphones and tablets, were perceived by medical experts as of equal or even better quality compared to when viewed on a computer screen. In addition, smartphones and tablets outperformed computers not only for electrocardiograms (ECGs) and X-rays, but also for clinical photographs of dermatological conditions and burn wounds.

#### Assessment of expected diagnostic accuracy – computer-based

In an additional study, the diagnostic accuracy determined by experts looking at burn images of various complexity on a computer screen was assessed [20]. Bedside diagnosis was used as a gold standard. It was observed that burns were diagnosed at least as well using photographs as at bedside and pediatric cases were significantly more accurately diagnosed than adult ones. By contrast, there were no differences between medical specialties or between countries of practice of physicians. Inter-rater reliability was higher for burn surgeons and for physicians from South Africa and intra-rater reliability was high (but only a few raters were considered).

#### Assessment of expected diagnostic accuracy – handheld devices

To move forward on the question of diagnostic accuracy a study is currently being conducted based on the type of burn injuries that are most likely to be seen in emergency care services in the Western Cape Province. Ten such scenarios/situations have been identified and 2–3 pictures have been selected as representative of each of them (all with a bedside diagnosis).

### Health impact

The ultimate goal of the system is to improve health outcomes of the patients. In the first step, we have focused on the short-term clinical impact of the system, but when the system has been used for a longer period of time it will obviously be interesting to also study the potential long-term impact of the system.

#### Choice of design to measure health impact

A historical cohort design will be applied to capture potential change in health outcomes of the patients after the introduction of the system. A historical cohort study is a longitudinal study of a group of individuals that share a common exposure (in this case use of diagnostic mHealth support) to determine its influence on health outcomes and compared to an equal group that have not been exposed. The data are being gathered through the medical records of the participating health facilities for a period of about one year (January 2017–February 2018).

#### Choice of acute health outcome measures

One essential aspect of the evaluation is to determine the relevant acute phase burn outcomes that may be affected by the mHealth system. Traditionally, mortality has been the most frequently used burn outcome measure and even if this is still the case, especially in some age groups, the improved survival after burn injuries has shifted the focus to long-term functional and health-related outcome measures [21–23].

The function of emergency care is to provide definitive care for those who do not need admission, or to help patients who are more seriously ill or injured transition safely into the hospital setting. Under those circumstances, the outcome of the patient should reflect the result of the definite care at the emergency department or an adequate referral to a specialized unit. Adequate referrals are therefore an important outcome measure to use in emergency care. A recent study from South Africa has also indicated that a high number of referrals changed when photographs were added to the ordinary telephonic referral [7].

Length of stay (LOS) is another burn outcome measure frequently used to assess the cost and quality of burn care. However, LOS is closely related to burn size and age and it has been suggested to use a ratio of hospital days per percentage Total Body Surface Area (TBSA) [20]. The ratio of LOS over %TBSA should be approximately 1, at least in high-resource settings. It has been debated whether the ratio can be used for minor burns involving hands or other functional body areas or for patients with social problems, but the consensus still suggests that LOS/%TBSA is a useful outcome measure [23]. Other outcome measures for acute burn care that have been proposed by the American Burn Association include psychological outcomes, burn resuscitation, nutritional and functional outcomes and burn wound-healing outcomes [24]. Some of these outcomes can be assessed in the acute phase and others need a longer follow-up after the burn to be assessed correctly. The current study will focus on the short-term health outcomes, but there is also a need to follow-up potential long-term health effects in future studies.

The primary outcome measure in the evaluation plan is defined as adequate referrals. A burns specialist with vast experience of burn care will define appropriate referral. The specialist will go through all patient files and decide whether or not each case should have been referred to a dedicated burns unit or not. The ratio of LOS over %TBSA will be included as the secondary outcome.

#### Data collection and data analysis to assess health impact

The data are retrospectively extracted by a research assistant from medical records at each hospital’s emergency department, including notes from the doctors, nurses and emergency medical services (EMS). Inclusion criteria will be burn injuries severe enough to remain at the hospital for at least one night or those being transferred to another health care facility. A standardized case report form based on World Health Organization (WHO) guidelines [25] will be used for the data collection on-site. Data will cover aspects relative to the injured patient (age, sex), the injury itself, some specific injury circumstances and information regarding referral. The data will be systematically collected on all injury cases to include TBSA, burn depth, Abbreviated Injury Scale (AIS), sex, mechanism of the burn and if the patient was transferred or treated and discharged.

Data will also be stored on the Vula Mobile cloud server. On any patient where the Vula app is used, their de-identified data, including all of the information mentioned earlier, will be stored in a secure password-protected cloud sever. Only the research team and a Vula administrator will have access to these data. Patients where the app is used will also need the data from their physical folders, as mentioned earlier, recorded as well.

The exposure of interest is use of diagnostic mHealth support. Patients with burn injuries where the doctor at point of care has used the app to receive diagnostic support from a burns expert will be considered as exposed. Patients with burn injuries where the app has not been used will be considered as non-exposed. Adequate referral assessed by a burns expert will be defined as the primary outcome. LOS per %TBSA will be considered as the secondary outcome.

A minimum sample size, based on the assumptions of a baseline rate of adequate referrals of 30% and about 50% exposure to the app, of 625 patients is required to show a difference of 10% referral rates between groups with 80% power (two-sided alpha = 0.05).

Logistic regression models will be used to investigate the effects of mHealth diagnostic support.

## Ethical considerations in relation to intervention and follow-up

The app follows the regulations and guidelines for development and application of health apps [26]. Anonymity of the images will be strived for but can be hard to achieve for patients with burns on the face or head. The images and clinical information will be treated as confidential, be assigned a unique identifier unrelated to any other patient identifiers, and will be stored on a secure cloud-based server which is accessed through a password-protected personal account for on-call teleexperts and for study personnel. The Vula app is now considered part of standard care in the Western Cape, so obtaining informed consent from the patients is not required. No data are held on the phones – all data are deleted automatically once the case is marked as resolved by the telexpert. Any data which are not successfully uploaded get remotely wiped after 24 hours. Teleexperts and treating clinicians only have access to the cases that they are directly responsible for.

Demographic and injury-related data, in addition to comorbidity and medication information, are collected on all burns patients presenting to the study sites on whom the mHealth system is not used. Patient identifiers are deleted and electronic data are stored on a password-protected work computer. Quality control of the data collection is done regularly. During the project, additional coding and recoding of the data are discussed within the research group and a logbook is kept so as to keep track of the various decisions taken along the way.

Only members of the research team have access to the key and password. All follow-up studies have received ethical approval from Stellenbosch University.

## Other methodological and ethical considerations

There are several issues that need to be considered in relation to the planned evaluation. Use of smartphones, tablets and mobile apps is part of many health care providers’ working life today and the use of WhatsApp and Instagram has increased exponentially over the last five years [27]. Nevertheless, some ethical considerations need to be mentioned related to the increased use of social media in the health sector, the most important being patient privacy and confidentiality when data is sent via the Internet. Another issue is that patient data stored on private smartphones will not be documented within the health system. A consultation system like the one described herein, on the other hand, can capture patient data and photographs in a protocol-driven manner within the health system.

The widespread use of social media among health care providers also causes methodological challenges in relation to the choice of an unexposed control group. Even front-line clinicians that have not used the app may have used other social media for diagnostic support, which makes them closer to those that have used the app. This will be carefully considered when selecting the control group and when collecting data on exposure.

Another challenge, when it comes to determining the health impact of the consultation system, is to identify outcome measures to detect meaningful changes in patient status that can be explained by the teleconsultation system. Adequate referrals have been identified as such and will be included as the primary outcome in the follow-up. A recent study from South Africa showed that two thirds of the referrals were changed when photographs were added to the ordinary telephonic referral [7]. The secondary outcome that will be included is LOS per %TBSA. This measure has been used in a recent study from South Africa [16], so even if it has been discussed that the ratio can be problematic for patients with social problems, it has previously been used in the same setting.

The accuracy of the system in relation to current practice and if the impact varies with type of burn or burn severity are also important issues that need to be taken into account. The accuracy may also change over time e.g. if regular use increases performance.

Despite these challenges and the few extra minutes it takes to use the app for clinical consultation, the system is expected to improve care delivery and facilitate local management whenever possible, which reduces stress of transport and long distances to travel for patients and caregivers. Additionally, care is likely to be enhanced through the timely addition of specialist advice and knowledge through the system.

## Conclusions

Smartphone-based consultation systems have the potential to increase real-time consultation to make more targeted and better-informed clinical decisions, but the evidence of their impact is still sparse, especially for acute conditions. Initial assessments within the current project showed that the quality of images taken at point of care with smartphone cameras is good enough to be used for diagnostic support. The results showed that both size and depth of burns could be assessed at least as well using photographs as at bedside and that the image quality of handheld devices can be used as well as computers [18,19]. There are several challenges that need to be considered when evaluating the potential health impact of the system. We suggest using a historical cohort design with adequate referrals and LOS per %TBSA as potential outcome measures.

## Paper context

Even though previous studies indicate that mobile phone-based applications for burns tend to support valid diagnosis and influence patient management, the evidence is still sparse. There is a need for more in-depth evaluations, not least in resource-constrained settings where mHealth projects are increasing. This article describes such a system for acute burns in the Western Cape, South Africa, assessments in relation to its implementation and the challenges of using image-based diagnostic support in the acute phase of burns.

## References

[1] Lewis ER, Thomas CA, Ph D, et al Telemedicine in acute-phase injury management: a review of practice and advancements. Telemed J E Health. 2011;18:434–48. 10.1089/tmj.2011.0199 PMC3399110 22694296

[2] Keane MG. A review of the role of telemedicine in the accident and emergency department. J Telemed Telecare. 2009;15:132–134. 19364895 10.1258/jtt.2009.003008

[3] Hasselberg M, Beer N, Blom L, et al Image-based medical expert teleconsultation in acute care of injuries. A systematic review of effects on information accuracy, diagnostic validity, clinical outcome, and user satisfaction. PLoS One. 2014;9:e98539. 10.1371/journal.pone.0098539 PMC4041890 24887257

[4] Saffle JR, Edelman L, Theurer L, et al Telemedicine evaluation of acute burns is accurate and cost-effective. J Trauma. 2009;67:358–365. 19667890 10.1097/TA.0b013e3181ae9b02

[5] Shokrollahi K, Sayed M, Dickson W, et al Mobile phones for the assessment of burns: we have the technology. Emerg Med J. 2007;24:753–755. 17954825 10.1136/emj.2007.046730 PMC2658315

[6] Atiyeh B, Dibo SA, Janom HH Telemedicine and burns. An overview. Ann Burns Fire Disasters. 2014;27:87–93. 26170782 PMC4396801

[7] Den Hollander D, Mars M Smart phones make smart referrals. The use of mobile phone technology in burn care – A retrospective case series. Burns. 2017;43:190–194. 27575675 10.1016/j.burns.2016.07.015

[8] Wallace DL, Hussain A, Khan N, et al A systematic review of the evidence for telemedicine in burn care: with a UK perspective. Burns. 2012;38:465–480. 22078804 10.1016/j.burns.2011.09.024

[9] WHO Fact sheet on burns. Reviewed 2016 9 [cited 2017 Feb 27]. Available from: http://www.who.int/mediacentre/factsheets/fs365/en/

[10] World Health Organization. A WHO plan for burn prevention and care World health organisation. Geneva: WHO Press, WHO; 2016.

[11] Kumar S, Nilsen WJ, Abernethy A, et al Mobile health technology evaluation. The mHealth evidence workshop. Am J Prev Med. 2013;45:228–236. 23867031 10.1016/j.amepre.2013.03.017 PMC3803146

[12] Scott RE, Saeed A Global eHealth – Measuring Out-comes: Why, What, and How. A report commissioned by the World Health Organization’s Global Observatory for Health. [cited 2017 3 13]. Available from: www.ehealth-connection.org

[13] South Africa Census 2011 [cited 2017 2 27]. Available from: https://census2011.adrianfrith.com/place/1

[14] Wallis LA, Fleming J, Hasselberg M, et al A smartphone app and cloud-based consultation system for burn injury emergency care. PLoS One. 2016;11:e0147253. 10.1371/journal.pone.0147253 PMC4769217 26918631

[15] Leavitt HJ Managerial psychology. Chicago: University of Chicago Press; 1972.

[16] Crumley Iona Remote Expert Diagnosis in Trauma and Emergency Medicine. Understanding Perspectives from the Tele-Experts in a Resource Constrained Setting, with Regards to Acute Burn Care. [Master thesis in Global Health]. Stockholm: Department of Public Health Sciences, Karolinska Institutet; 2015.

[17] Yean P-Y Health Information Technology usability Evaluation: Methods, Models and Measures. 2010 [cited 2 27]. Available from: http://gradworks.umi.com/34/20/3420882.html

[18] Boissin C, Fleming J, Wallis L, et al Can we trust the use of smartphone cameras in clinical practice? Laypeople assessment of their image quality. Telemed E-Health. 2015;21:887–892. 10.1089/tmj.2014.0221 PMC4649724 26076033

[19] Boissin C, Blom L, Wallis L, et al Image-based teleconsultation using smartphones or tablets: qualitative assessment of medical experts. Emerg Med J. 2017;34:95–99. 27707791 10.1136/emermed-2015-205258 PMC5384429

[20] Boissin C, Laflamme L, Wallis L, et al Photograph-based diagnosis of burns in patients with dark-skin types: the importance of case and assessor characteristics. Burns. 2015;41:1253–1260. 25716764 10.1016/j.burns.2014.12.014

[21] Palmieri TL, Przkora R, Meyer WJ, et al Measuring burn injury outcomes. Surg Clin N Am. 2014;94:909–916. 25085096 10.1016/j.suc.2014.05.010

[22] Staley M, Richard R, Warden G, et al Functional outcomes for the patient with burn injuries. J Burn Care Rehabil. 1996;17:362–368. 8844359 10.1097/00004630-199607000-00014

[23] Allorto NL, Clarke DL Merits and challenges in the development of a dedicated burn service at a regional hospital in South Africa. Burns. 2015;41:454–461. 25149190 10.1016/j.burns.2014.07.021

[24] Gibran NS, Wiechman S, Meyer W, et al Summary of the 2012 ABA burn quality consensus conference. J Burn Care Res. 2013;34:361–385. 23835626 10.1097/BCR.0b013e31828cb249

[25] Holder Y, Peden M, Krug E, et al Eds. Injury surveillance guidelines. Geneva: World Health Organization; 2001.

[26] Kamel Boulos MN, Giustini DM, Wheeler S Instagram and WhatsApp in health and healthcare: an overview. Future. 2016;8:37.

[27] Mars M, Scott RE WhatsApp in clinical practice: a literature review. IOS Press; 2016. 27782019

